# Evaluation of India’s Supplementary Nutrition Programme for children aged 36–72 months and opportunities for improvement using linear programming

**DOI:** 10.1017/jns.2026.10095

**Published:** 2026-05-22

**Authors:** Ankita Mondal, Jawahar R. Manivannan, Gowri Bhatnagar, Fathima Ayoob, Ashikh Ahamed, Afsal K. Murikkancheri, Melari S. Nongrum, Sandra Albert, Pulkit Mathur, Lalita Verma, Radhika S. Madhari, Srirangam A. Brinda, Suparna Ghosh-Jerath, Vanisha Nambiar, Hemangini Gandhi, Syed Z. Quazi, Alka Mahobia, Rachita Gupta, Harshpal Singh Sachdev, Anura V. Kurpad, Tinku Thomas

**Affiliations:** 1 Division of Epidemiology and Biostatistics, St John’s Research Institute, Bengaluru, India; 2 Department of Public Health Nutrition, Indian Institute of Public Health Shillong, India; 3 Department of Food and Nutrition and Food Technology, Lady Irwin College, India; 4 Division of Dietetics, National Institute of Nutrition (ICMR), Hyderabad, India; 5 Department of Nutrition, The George Institute for Global Health India, India; 6 Department of Foods and Nutrition, MS University of Baroda, India; 7 Datta Meghe Institute of Medical Sciences, India; 8 Datta Meghe Institute of Higher Education and Research Deemed to be University, India; 9 World Health Organization Regional Office for South-East Asia, India; 10 Sita Ram Bhartia Institute of Science and Research, India; 11 St John’s Research Institute. St John’s National Academy of Health Sciences, India; 12 Department of Biostatistics, St John’s Medical College, India

**Keywords:** Anganwadi centres, Dietary diversity, Hot cooked meals, India, Integrated Child Development Services, Linear programming, Micronutrient deficiencies, Nutrient adequacy, Preschool children, Supplementary Nutrition Programme

## Abstract

India’s Supplementary Nutrition Programme (SNP), under the Integrated Child Development Services, provides a Morning Snack and a Hot Cooked Meal to children aged 36–72 months through Anganwadis (day-care centres). This study assessed these meals against SNP standards (2012) and age-specific ICMR recommendations, when standards were unavailable, and explored the use of linear programming (LP) to improve nutrient quality of SNP meals. A cross-sectional survey documented the SNP-meals, ingredients, serving portions and other details, using questionnaires administered to Anganwadi workers and programme officials from purposively selected Anganwadis across 27 States and Union Territories. Nutrient composition was estimated using standard food composition tables, and State-specific food lists and retail prices were incorporated into the LP framework to identify foods that could improve nutrient content of SNP meals. Energy standards were met in 56% of States, while 22% fell more than 20% below recommendations. Protein standards were achieved in 74% of States; however, declined to 52% after adjusting for digestibility. Only 22% of States met the ICMR-based fat requirement. Zinc, iron and folate were largely met, whereas calcium and vitamins A, B_6_ and B_12_ were below recommendations in more than half the States. Meals were predominantly cereal-based with limited inclusion of nutrient-dense foods. LP identified foods that reduced nutrient gaps, although many improvements exceeded the per-child cost allocation. Overall, meals showed wide variability, with persistent gaps in fat and key micronutrients. LP provides a structured approach to enhance nutrient provision within the programme setting, although meeting all nutrient targets may require adjustments to cost and procurement strategies.

## Introduction

After independence, India confronted widespread poverty, food insecurity, and persistent malnutrition, prompting the Government of India (GOI) to establish Integrated Child Development Services (ICDS) in 1975. The programme delivers an integrated package of early childhood care, health services and supplementary nutrition through a nationwide network of Anganwadis.^(1)^ Within ICDS, the Supplementary Nutrition Programme (SNP) provides a Morning Snack (MS) and a Hot Cooked Meal (HCM) for children aged 36–72 months, while younger children (6–36 months) receive Take-Home Rations.^(1)^

ICDS is among the world’s largest community-based feeding programmes, reaching nearly 36 million preschool-aged children and 90 million beneficiaries through 1.4 million Anganwadis across India.^(2,3)^ The programme is overseen nationally by the Ministry of Women & Child Development, which issues guidelines and funding norms, while States (administrative units within India) are responsible for implementation and adapt menus according to local conditions. In recent years, fortified staples such as rice and edible oil have been incorporated into the meals provided through ICDS as part of India’s national nutrition programme, POSHAN 2.0.^(4)^

The nutritional standards for meals provided to 36–72 month old children have evolved over time, increasing from 300 kcal and 8–10 g protein per child per day until 2009, and then to 500 kcal and 12–15 g protein until 2023.^(5,6)^ The 2023 revision marked a major policy shift by introducing recommendations for fat and key micronutrients (iron, calcium, zinc, vitamins A and B_12_), improving protein quality, and encouraging dietary diversity through the inclusion of locally available foods.^(7)^ These expanded standards were introduced without any increase in the per-child allocation of ₹8 per day, creating practical challenges in delivering nutrient-dense meals within fixed financial resources.

Linear programming (LP) is a mathematical optimisation method that identifies cost-efficient combinations of foods to meet specified nutrient targets within constraints such as budget and food availability. Although LP has been widely applied in dietary modelling for children and maternal diets and in small-scale menu planning studies,^(8,9,10)^ its use within large public feeding programmes such as the ICDS-SNP has been limited. Using LP in this context provides a structured approach to designing context-specific diets that meet programme nutrient standards within existing operational and financial boundaries.

Comprehensive evidence on the nutrient composition of foods provided under SNP is limited, with most assessments restricted to individual States.^(11,12)^ Understanding the nutrient provision relative to standards is essential for documenting how current menus align with programme standards and for generating evidence to support future menu planning under the revised 2023 nutrient standards. For the present analysis, SNP meals refer to all foods provided to children aged 36–72 months at the Anganwadis, during programme hours, encompassing the HCM and any additional food items provided that day under the SNP.

This study therefore assessed the nutritional composition of SNP meals across 27 States and Union Territories in India and applied LP-based optimisation to explore its potential application within the programme for designing nutrient-adequate menus using locally available foods and within existing budgetary limits. The specific objectives were:

- to assess the nutrient content of foods provided to children aged 36–72 months under the SNP across 27 States and Union Territories of India
- to compare the nutrient content of these foods with ICDS-SNP programme standards for energy and protein (2012), and with ICMR recommendations for fat and essential micronutrients
- to apply a linear programming framework to identify locally available, low-cost food combinations that could improve the nutrient quality of the SNP meals.

## Materials and methods

### Study design

A cross-sectional survey was conducted between August 2022 and February 2023 to document all foods served to children aged 36–72 months, attending Anganwadis during programme hours. Data were collected across multiple geographic regions of India. In total, data were analysed from 27 geographies (25 States and 2 Union Territories) (Supplementary Table S1), representing a broad cross-section of India’s 28 States and 8 Union Territories.

In the States of Punjab and Uttarakhand, HCM had ceased post-Covid pandemic, however, pre-pandemic menus were available and included in the analysis. In Rajasthan and Uttar Pradesh, HCM distribution had also ceased, however, pre-pandemic data were unavailable. In West Bengal, data could not be collected from the Anganwadis due to lack of administrative approval. Two Union Territories (federally administered regions) of Jammu & Kashmir and the National Capital Territory of Delhi, whose geographical and population sizes are comparable to those of States, were also included. All these administrative units will be collectively referred to as ‘States’ henceforth in this manuscript.

This study was funded by the World Health Organization (India Office; Grant Number 202868487-1) and conducted according to the principles of the Declaration of Helsinki. Ethical approval for the study was obtained from the Institutional Ethics Committee of St. John’s Medical College and Hospital, Bengaluru, India, and the study adhered to national ethical requirements for research involving human participants. Written informed consent was obtained from all participating Anganwadi workers and programme officials.

### Sampling framework

In each State, four Anganwadis were selected from two purposively sampled districts (two Anganwadis per district) based on logistical feasibility. Anganwadis with at least 10 beneficiary children (36–72 months) receiving meals prepared at the Anganwadi were eligible for inclusion.

In the NCT of Delhi, meals were sourced from centralised kitchens rather than prepared on-site, and therefore two central kitchens that supplied the four sampled Anganwadis were also selected. Anganwadis located in State capitals were excluded to avoid potential bias from better-resourced facilities, which may not reflect general practices in the rest of the State.

The sampling approach was designed to capture geographic variation in meal composition across States within available resources. The final sample included 108 Anganwadis across 27 States and Union Territories, representing a broad geographic and dietary diversity across India.

### Data collection

Data were collected using an electronic data-capture system developed for the study, with pre-tested and internally validated questionnaires translated into local languages. Information on Anganwadi characteristics, beneficiary categories and foods provided was obtained from the Anganwadi worker responsible for meal preparation and serving.

The menus, based on repeated meal plans (typically 6–15-day cycles), were recorded using a modified multi-pass recall method,^(13)^ capturing all foods cooked and served on each operational day of the week. Portion sizes of cooked food served per child were recorded and verified through direct observation during one to three site visits.

Additional information on food fortification, procurement, prices, and additional food provided through other safety-net programmes was obtained from the Supervisor or Child Development Project Officer, a block-level ICDS official overseeing a cluster of Anganwadis. Market prices of all food items used in meal preparation were recorded from three local vendors in local retail markets, and the mean price was used for linear programming.

### Data management and quality control

Real-time monitoring and quality checks of data were undertaken throughout the data collection period by a central coordination team. Six collaborating institutes across different geographic regions implemented the fieldwork. Field investigators from each institute were trained to ensure uniformity in data collection and accuracy in portion estimation.

### Data analysis

The nutrient content of raw foods was estimated using the Indian Food Composition Table (IFCT, 2017).^(14)^ Nutrient values for items not listed in the IFCT were sourced from the USDA Food Composition Database^(15)^ and adjusted for moisture content to ensure comparability. Vitamin B_12_ values, which are not reported in IFCT, were also obtained from USDA sources after moisture correction.^(15)^

State-wise means and variances of each macro- and micronutrient provided to a child through the SNP meals were first calculated. These summary statistics were then used to estimate the corresponding log-normal parameters, based on the assumption that nutrient intake follows a log-normal distribution. Using these estimated parameters, simulated nutrient intake values were generated from a log-normal distribution for each State. The lognormal distribution was chosen because it is a continuous probability distribution bounded between zero and positive infinity, which effectively accommodates the skewed (asymmetric) nature typically observed in nutrient intake data.^(16)^ A total of 1000 iterations of nutrient provision were created to represent the varying nutrient contents of foods provided to children through SNP meals. The resulting median and inter-quartile range (IQR) from these simulated values represent the daily nutrient provision for each State.

The daily nutrients provided through SNP meals, obtained through simulation, was compared against the ICDS-SNP standards (2012) for energy and protein for children aged 36–72 months, and ICMR age-specific recommendations for fat and essential micronutrients, which were not part of the SNP standards at the time of data collection (Supplementary Table S2). The resulting median and IQR of the simulated data represent the daily nutrients provided in each State (Table 1). The same dataset was re-evaluated against the revised 2023 standards (Supplementary Table S3).

**Table 1.** Daily nutrient content of foods provided through the ICDS-Supplementary Nutrition Programme to children aged 36–72 months across selected Anganwadi centres in 27 States and Union Territories in India, evaluated against the 2012 nutrient standards (Median (IQR))

State	Energy^a^ (kcal)	Protein^b^ (g)	Fat^c^ (g)	Calcium^d^ (mg)	Zinc^d^ (mg)	Iron^d^ (mg)	Folate^d^ (µg)	Vit A (RAE)^d^ (µg)	Vit B6^d^ (mg)	Vit B12^d^ (µg)
Andhra Pradesh^efg^	505.31 (100.04)	15.27 (1.99)	13.37 (7.71)	277.45 (118.42)	2.3 (0.37)	5.89 (2.34)	54.66 (33.43)	318.11 (263.2)	0.3 (0.1)	0.82 (0.14)
Arunachal Pradesh^e^	482.52 (93.61)	13.56 (5.21)	6.98 (5.65)	37.24 (35.37)	2.18 (0.84)	6.48 (1.72)	34.77 (42.33)	4.9 (6.28)	0.19 (0.09)	0.1 (0.01)
Assam^e^	676.62 (71.55)	16.79 (5.18)	17.48 (3.13)	171.16 (44.98)	2.69 (0.8)	7.15 (2.24)	52.01 (32.51)	25.36 (41.97)	0.22 (0.07)	0.4 (0.24)
Bihar	519.18 (106.09)	17.97 (4.84)	11.97 (6.54)	101.94 (49.65)	2.8 (0.72)	4.86 (1.8)	124.13 (53.88)	30.94 (30.13)	0.34 (0.13)	0.0 (0.0)
Chhattisgarh^eg^	416.74 (130.43)	12.35 (7.73)	8.11 (9.54)	75.37 (82.97)	1.84 (1.14)	6.26 (4.62)	70.68 (65.73)	39.41 (66.77)	0.17 (0.12)	0.04 (0.01)
Goa	636.25 (135.32)	20.59 (5.48)	33.62 (15.28)	198.88 (131.71)	3.41 (1.26)	7.06 (5.66)	114.25 (49.81)	3.12 (2.22)	0.36 (0.13)	0.0 (0.0)
Gujarat	498.73 (150.13)	11.75 (4.41)	14.31 (7.21)	52.46 (28.9)	2.57 (0.92)	3.91 (1.74)	43.48 (34.13)	18.66 (28.71)	0.32 (0.09)	0.0 (0.0)
Haryana^e^	622.87 (209.28)	15.61 (5.91)	16.24 (10.15)	79.91 (45.17)	3.01 (1.21)	6.36 (2.25)	89.09 (58.06)	35.79 (44.11)	0.32 (0.13)	0.02 (0.03)
Himachal Pradesh	469.09 (117.97)	10.61 (4.12)	19.11 (10.85)	84.56 (49.28)	1.55 (0.6)	2.74 (1.02)	57.69 (39.26)	8.92 (12.82)	0.17 (0.09)	0.0 (0.0)
Jammu & Kashmir^e^	510.76 (100.42)	10.48 (0.7)	9.67 (7.61)	23.86 (13.92)	1.67 (0.07)	4.77 (0.77)	20.78 (17.82)	1.19 (1.02)	0.15 (0.03)	0.07 (0.02)
Jharkhand	500.95 (227.73)	17.19 (7.15)	9.8 (4.01)	55.33 (28.49)	2.64 (1.13)	4.46 (1.92)	56.7 (37.37)	58.39 (27.57)	0.29 (0.12)	0.0 (0.0)
Karnataka^ef^	425.21 (84.03)	13.02 (3.13)	9.94 (5.01)	90.67 (70.34)	1.84 (0.45)	4.56 (1.4)	38.58 (23.74)	64.54 (50.31)	0.25 (0.07)	0.23 (0.07)
Kerala^e^	494.61 (136.27)	14.14 (4.76)	9.5 (6.49)	83.81 (50.71)	2.11 (0.83)	3.57 (1.43)	62.25 (24.59)	81.43 (129.46)	0.33 (0.13)	0.02 (0.05)
Madhya Pradesh	347 (62.37)	10.13 (2.16)	7.63 (5.06)	52.76 (53.47)	1.74 (0.29)	3.12 (1.1)	41.23 (26.87)	10.21 (9.5)	0.2 (0.09)	0.03 (0.06)
Maharashtra	399.6 (68.87)	11.7 (2.14)	16.39 (4.32)	61.99 (8.79)	1.86 (0.24)	4.24 (0.7)	80.18 (11.62)	27.38 (5.51)	0.24 (0.03)	0 (0)
Manipur	465.5 (231.31)	15.44 (4.3)	11.49 (8.97)	315.54 (160.71)	2.00 (0.58)	4.01 (3.5)	68.01 (27.1)	93.78 (113.06)	0.19 (0.08)	0.4 (0)
Meghalaya^e^	598.52 (110.24)	20.42 (4.66)	3.95 (1.73)	281.79 (34.74)	2.36 (0.88)	10.75 (3.05)	74.62 (48.61)	125.98 (7.61)	0.21 (0.09)	0.02 (0.03)
Mizoram^efg^	524.28 (306.01)	14.31 (7.71)	24.09 (15.39)	179.19 (211.65)	1.5 (0.66)	9.56 (7.3)	52.17 (36.25)	164.04 (157.01)	0.14 (0.08)	0.21 (0.37)
NCT of Delhi	366.45 (202.03)	12.45 (7.04)	11.74 (7.63)	59.56 (44.48)	2.06 (1.1)	3.76 (2.2)	85.29 (75.51)	28.08 (30.68)	0.21 (0.13)	0.0 (0.0)
Nagaland	215.5 (128.24)	4.91 (5.47)	3.03 (3.34)	11.85 (10.54)	0.81 (0.94)	1.21 (1.4)	9.54 (11.43)	0.64 (1.2)	0.07 (0.06)	0.0 (0.0)
Odisha^e^	522.4 (84.64)	15.39 (3.64)	6.04 (4.41)	58.48 (26.31)	2.27 (0.47)	6.08 (0.97)	56.82 (26.77)	19.72 (17.08)	0.35 (0.08)	0.23 (0.16)
Punjab	458.76 (110.13)	10.7 (5.0)	8.12 (4.22)	158.95 (98.31)	1.95 (0.59)	1.73 (1.39)	13.3 (8.51)	0.07 (0.08)	0.21 (0.08)	0.0 (0.0)
Sikkim	457.33 (153.62)	14.76 (7.34)	7.79 (6.34)	159.66 (171.56)	2.3 (1.22)	3.78 (2.47)	87.71 (45.08)	13.8 (16.91)	0.38 (0.32)	0.38 (0.5)
Tamil Nadu^f g^	506.92 (51.37)	13.49 (3.13)	9.45 (2.1)	103.64 (28.5)	2.55 (0.37)	4.98 (1)	59.6 (20.35)	78.01 (79.8)	0.48 (0.11)	0.04 (0.08)
Telangana^fg^	374.59 (125.38)	10.36 (4.02)	8.13 (5.39)	27.78 (52.74)	1.42 (0.78)	1.63 (1.66)	40.24 (34.59)	43.63 (82.57)	0.16 (0.07)	0.03 (0.06)
Tripura^e^	263.93 (74.69)	9.91 (5.03)	1.19 (1.37)	31.76 (30.98)	1.59 (0.94)	3.8 (1.99)	38.44 (48.11)	1.63 (2.43)	0.15 (0.09)	0.06 (0.08)
Uttarakhand	304.46 (191.15)	13.77 (9.63)	3.16 (4.87)	51.03 (43.92)	2.17 (1.56)	4.28 (3.14)	65.34 (54.69)	8.16 (13.31)	0.26 (0.21)	0.0 (0.0)
Pooled	465.3	13.6	11.2	106.9	2.12	4.85	58.95	48.37	0.25	0.11
ICDS (old)*	500	12	13.8–19.4	150	1.2	2.6	37	80	0.3	0.7
Difference	−34.7	1.6	−2.6	−43.1	0.9	2.3	21.9	−31.6	−0.05	−0.59

The values represent the median (IQR) of nutrients provided through SNP meals to each child aged 36–72 months during programme hours. Colours indicate the percentage of requirements met for each nutrient: green denotes ≥90% of the requirement, yellow denotes 80–90%, and red denotes <80%. For energy and fat, red also indicates provision exceeding 10% above the recommendation.

a Energy is considered met if ≥90% of the ICDS norm of 500 kcal is provided.

b Protein is considered met if the minimum ICDS standard of 12 g/day is provided.

c Fat is considered met when the ICMR recommendation of 25–35% fat to energy ratio is achieved, with a 10% allowance.

d For micronutrients, requirements are considered met if meals provided one-third of the age-specific Estimated Average Requirement (EAR) for children 3–6 years, with a 10% tolerance.

e Anganwadis surveyed in these States used rice fortified with iron (4.25 mg/100g), folic acid (12.5 µg/100g) and vitamin B_12_ (0.125 µg/100g).

f Anganwadis surveyed in these States used wheat flour fortified with iron (4.25 mg/100g), folic acid (12.5 µg/100g) and vitamin B_12_ (0.125 µg/100g).

g Anganwadis surveyed in these States used Vitamin A-fortified oil (750 µg/litre) in meal preparation.

Energy content of SNP meals was considered adequate if they provided at least 90% of the SNP standard of 500 kcal. Protein was evaluated against the lower bound of the programme standard (12 g/day). Protein content was then adjusted using a digestibility factor of 0.8, as recommended for predominantly cereal-based diets in India,^(17)^ and re-evaluated. The fat requirement was set at the ICMR-recommended fat-to-energy ratio of 25–35%. For micronutrients, adequacy was defined as provision of one-third of the age-specific Estimated Average Requirement (EAR), as recommended by ICMR^(18)^ (Supplementary Table S2), considering that the SNP meals are expected to provide at least one-third of the daily nutrient needs. A 10% flexibility margin was applied to both macro- and micronutrients when assessing adequacy. Fortified ingredients provided through SNP and used in meal preparation were included in nutrient calculations, while foods supplied through other government initiatives were excluded.

A linear programming framework was applied to identify combinations of locally available, cost-efficient foods that could improve nutrient content within the ICDS-SNP standards and budgetary constraints. State-specific food lists and local retail prices were used. The existing ₹8 (INR) per-child daily allocation was used as the cost reference in the optimisation. Details of the optimisation framework, parameters, and constraints are provided elsewhere.^(19)^

## Results

The analysis included data from 108 Anganwadis across 27 Indian States and Union Territories, collected between August 2022 and February 2023.

The nutrient content of the SNP meals varied substantially across States (Table 1). The energy content met the programme standard in 15 States, was more than 20% below the standards in six States, and exceeded the standard by more than 20% in four States. Protein content met the standard in 74% of States; however, after applying 80% digestibility correction, only 52% met the required protein level.^(6)^ The proportion of energy derived from fat fell within the recommended 25–35% range in six States, fell below this range in most States, and exceeded 35% in three. For micronutrients, zinc (96%), iron (89%), and folate (89%) most consistently met the recommended values. In contrast, calcium, vitamin A, vitamin B_6_, and vitamin B_12_ contents showed wide variability. Calcium content met the standards in eight States, vitamin A in six, and vitamin B_6_ in ten States. Vitamin B_12_ was the most limiting nutrient, with only one State meeting the required level.

State-wise contributions of each food group to total energy and protein are presented in Table 2. Across States, cereals and millets remained the largest contributors to total energy (38–77%) and protein (35–74%). Pulses and legumes contributed 7–25% of energy and 18–54% of protein, while milk and eggs contributed up to 12% when included. Contributions from vegetables, fruits, and other non-staple foods were minimal across most settings.

**Table 2.** Contribution of major food groups (%) to energy and protein in meals provided through the ICDS-supplementary nutrition programme to children aged 36–72 months in India

States	Cereals and Millets (%)		Pulses and legumes (%)		Eggs and other non-veg products (%)		Milk and milk-based products (%)		Fruits and Vegetables (%)		Edible Oils and Fats (%)		Sugars* (%)		Others (includes nuts and seeds) (%)	
	E	P	E	P	E	P	E	P	E	P	E	P	E	P	E	P
Andhra Pradesh	49	30	9	16	6	17	16	23	3	6	8	0	3	0	5	6
Arunachal Pradesh	64	51	18	45	0	0	0	0	1	1	14	0	0	0	0	0
Assam	37	38	8	30	0	4	9	15	6	4	5	0	28	0	7	7
Bihar	42	31	13	30	0	0	11	19	2	4	7	0	13	2	6	10
Chhattisgarh	39	24	21	43	0	0	0	0	8	20	13	0	8	0	8	10
Goa	24	17	15	30	0	0	0	0	7	19	24	0	8	1	23	33
Gujarat	41	43	21	44	0	0	0	0	2	5	16	0	13	2	2	2
Haryana	41	44	8	28	0	0	0	0	1	5	15	0	21	0	9	17
Himachal Pradesh	37	38	9	35	0	0	6	14	1	2	18	0	13	0	15	8
Jammu & Kashmir	55	63	9	34	0	0	0	0	0	0	12	0	22	0	2	2
Jharkhand	31	21	24	49	0	0	0	0	3	3	10	0	8	0	16	21
Karnataka	42	27	8	17	7	21	16	24	1	2	9	0	9	0	6	8
Kerala	21	16	13	26	13	38	0	0	3	5	5	0	21	3	22	10
Madhya Pradesh	35	36	7	14	0	0	15	22	10	25	19	0	10	0	1	1
Maharashtra	35	32	27	62	0	0	0	0	1	2	23	0	11	0	2	3
Manipur	28	20	17	36	0	0	12	24	2	5	18	0	11	0	11	13
Meghalaya	79	62	12	36	0	0	0	0	0	1	4	0	4	0	0	0
Mizoram	17	10	9	16	7	17	28	33	0	0	17	0	2	0	21	25
Nagaland	60	44	22	55	0	0	0	0	0	0	18	0	0	0	1	1
NCT of Delhi	25	25	20	46	0	0	0	0	10	13	16	0	14	0	10	12
Odisha	23	13	25	42	12	28	0	0	1	1	9	0	12	2	7	8
Punjab	46	61	0	0	0	0	15	39	0	0	19	0	19	0	0	0
Rajasthan	52	59	13	41	0	0	0	0	0	0	35	0	0	0	0	0
Sikkim	68	56	3	10	0	0	17	29	2	4	4	0	5	0	1	1
Tamil Nadu	33	25	10	23	12	36	0	0	4	8	6	0	33	6	0	0
Telangana	67	46	12	25	8	26	0	0	2	3	10	0	0	0	0	0
Tripura	48	23	24	36	15	32	0	0	2	6	0	0	4	0	0	0
Uttarakhand	28	36	11	40	0	0	0	0	13	16	13	0	28	0	7	6

E = Energy; P = Protein.

The table presents the percentage contribution of each major food group to total energy and protein provided through SNP meals during programme hours.

* The protein contribution observed under the “Sugars” food group is attributable to the inclusion of jaggery in meals provided by the selected Anganwadi centres in the respective States. According to the Indian Food Composition Tables (IFCT), jaggery contains small amounts of protein.^(14)^

The updated standards lowered the energy target (from 500 kcal to 400 kcal), while increasing protein and fat requirements and introduced micronutrient benchmarks. While most States met the lower energy requirement, shortfalls in protein, fat, calcium, vitamin A, vitamin B_6_ and vitamin B_12_ persisted, indicating a need for more nutrient-dense combinations within operational constraints.

The optimisation exercise was carried out for three States, including Karnataka, Madhya Pradesh and Maharashtra, selected as examples to illustrate diverse geographic and dietary contexts. Results indicated that small additions of locally available nutrient-dense foods such as eggs, milk, green leafy vegetables and other vegetables substantially reduced nutrient gaps. However, these improvements exceeded the current ₹8 (INR) per-child daily allocation, requiring an additional ₹8 (INR) in Karnataka and Maharashtra and ₹12 (INR) in Madhya Pradesh (Supplementary Tables S4a–S4c).

## Discussion

The meals provided to children aged 36–72 months under the SNP generally met the programme standards for energy and protein, primarily through cereals and pulses. However, when protein quality was accounted for, the meals did not meet the recommended protein standard for nearly half the States. A previous assessment of HCM reported that it provided around 78% of the intended energy and protein for pre-school children.^(11)^ In the present analysis, the meals also showed consistent shortfalls in fat and several essential micronutrients. To our knowledge, fat and micronutrient content of meals provided to children aged 36–72 months under the SNP has not been previously assessed. However, evidence from Take-Home Rations for younger ICDS beneficiaries shows similar gaps, with deficits in fat, iron, calcium, zinc, folate and vitamins A and B_12._^(20)^

The predominance of cereals in the meals reflects broader dietary patterns in India, where monotonous cereal-based meals with limited nutrient-dense foods have been linked to widespread micronutrient deficiencies.^(21)^ Biomarker findings from the Comprehensive National Nutrition Survey of Children, show that deficiencies of zinc, iron, folate, vitamin B_12_ and vitamin A remain common among 1–4 year old children, underscoring the broader nutritional challenges to which the ICDS-SNP is expected to contribute.^(22)^ Consistent with previous studies,^(23)^ the present study also found the SNP meals to be predominantly cereal-based, highlighting the need to improve its nutrient density through locally available, nutrient-dense foods.

The 2023 revision of SNP standards marks a shift toward nutrient density, increasing the protein target to 15–20 g, introducing a 15–20 g fat requirement and adding benchmarks for essential micronutrients, while reducing the energy norm from 500 kcal to 400 kcal. Although the meals in this study were collected under the earlier standards, supplementary comparison with the updated standards (Supplementary Table S3) indicates that the higher protein, fat and micronutrient requirements amplify the existing gaps. Meeting these revised standards will require higher nutrient density within comparable energy and budgetary limits. This is particularly pertinent for young children, whose limited gastric capacity constrains food volume and necessitates more nutrient-dense meals.^(24)^

Emerging evidence shows that a considerable proportion of Indian children experience a dual burden, with anthropometric undernutrition occurring alongside early metabolic abnormalities.^(25)^ As India moves through a nutrition transition, characterised by limited dietary diversity and increased metabolic risk, avoiding excess energy intake remains important, since even small daily surpluses can contribute to a median annual weight gain of 0.5 to 1 kg.^(26,27)^ Thus optimising SNP menus can support ‘double-duty action’ by improving micronutrient adequacy while maintaining a balanced energy-protein-fat provision, thereby helping reduce both undernutrition, as well as addressing the emerging risk of overweight in early childhood. Together, these considerations highlight the need for systematic approaches to menu design that can improve dietary quality while balancing operational constraints.^(28)^

In this study, the linear programming framework was applied as an exploratory tool to demonstrate its utility within the ICDS context, identifying combinations of locally available foods using State-specific food lists and market prices that could improve nutrient density under prevailing constraints. These outputs showed that modest additions of nutrient-dense foods such as eggs, milk, green leafy vegetables and other vegetables substantially improved multiple micronutrients simultaneously. These findings align with earlier applications of linear programming in developing complementary foods, where the approach helped identify limiting nutrients, and formulate low-cost food combinations across diverse settings.^(9,24,29,30)^ Importantly, optimisation frameworks operate within the foods and prices that are realistically accessible, making them well suited for programme environments where cost, availability and cultural acceptability are central considerations.^(10,30)^ In the present analysis, the foods contributing most to reducing nutrient gaps were also those with higher market prices, which explains why even small additions resulted in costs exceeding the current ₹8 (INR) per-child allocation. This aligns with a broader challenge from low-and middle-income countries where micronutrient-dense foods are often relatively more costly than staple cereals.^(31)^

The optimisation framework used in this study has been translated into an easy-to-use interactive tool (www.datatools.sjri.res.in), that enables programme officials to examine how alternative food combinations affect nutrient provision and cost using State-specific food lists and prices.^(19)^ The tool is adaptable to diverse settings and has been configured to incorporate the 2023 SNP nutrient standards. When interpreted in conjunction with local preferences, supply considerations and food preparation capacity, such tools can support more informed and context-appropriate decisions for menu planning under the revised nutrient standards.

Practical implementation will, however, depend on local programme conditions. Many of the foods that most effectively improve nutrient provision, such as vegetables, green leafy vegetables, milk and eggs, require reliable procurement systems, stable supply chains and adequate storage and preparation capacity at Anganwadis. The optimisation tool can support States to navigate these constraints by identifying ‘best feasible’ menu options when fully optimal combinations are not practical due to procurement challenges. In settings with limited market access or higher procurement costs, complementary approaches such as Anganwadi or community kitchen gardens can help increase access to green leafy vegetables and other vegetables, improving dietary diversity without substantial additional expenditure.^(32,33)^

This study has several limitations. The sample of Anganwadis was purposively selected based on operational feasibility and, although it spans 27 States and Union Territories, it does not constitute national coverage. The absence of SNP meal data from Rajasthan, Uttar Pradesh and West Bengal further limits geographic representation. The meals captured were those in use at the time of the survey, and may not reflect seasonal or district-level variation in food availability or procurement. Despite these limitations, the study provides a broad multi-State perspective on the ICDS-SNP for children aged 36–72 months and offers empirical insights into nutrient gaps and opportunities for strengthening menu design.

## Conclusion

This study assessed the nutritional quality of meals provided under the ICDS-SNP across diverse Indian settings and identified consistent gaps in fat and key micronutrients, despite generally adequate provision of energy and protein. These findings highlight the need to improve nutrient density within the programme, particularly in light of the revised 2023 SNP standards, which place greater emphasis on dietary quality.

The study demonstrates the potential of linear programming to identify locally feasible food combinations that improve nutrient provision within defined constraints. The optimisation framework has been translated into an accessible interactive tool that enables programme officials to explore how different food choices influence both cost and nutrient provision using State-specific food lists and prices. When used alongside complementary strategies such as improved procurement mechanisms and locally feasible sources of green leafy vegetables and other vegetables, this approach may support improvements in the nutritional quality of meals delivered through ICDS-SNP.

## Supporting information

10.1017/jns.2026.10095.sm001 Mondal et al. supplementary material Mondal et al. supplementary material

## Data Availability

The data described in the manuscript will be made available upon request to the corresponding author.

## Supplementary material

The supplementary material for this article can be found at https://doi.org/10.1017/jns.2026.10095.
