# Supplementary material for: Evaluation of India’s Supplementary Nutrition Programme for children aged 36–72 months and opportunities for improvement using linear programming
Source: J Nutr Sci. 2026 May 22;15:e36. doi: 10.1017/jns.2026.10095 (PMC13200022; doi:10.1017/jns.2026.10095)
Supplement: Mondal et al. supplementary material [file S2048679026100950sup001.docx]

**Supplementary Table S1:** Geographic coverage of the survey across Indian States and Union Territories under the Integrated Child Development Services (ICDS)

| **Region** | **State / Union Territory** | **Districts Surveyed** |
| --- | --- | --- |
| **North** | Jammu & Kashmir | Srinagar; Ganderbal |
|  | Punjab | Hoshiarpur; Amritsar |
|  | Haryana | Kurukshetra; Rewari |
|  | Himachal Pradesh | Kangra; Kullu |
|  | Uttarakhand | Hardwar; Nainital |
|  | NCT of Delhi | South-West Delhi; Central Delhi |
| **East** | Bihar | Munger; Patna |
|  | Odisha | Khordha; Rayagada |
|  | Jharkhand | Pashchimi Singhbhum; Ramgarh |
| **North-East** | Arunachal Pradesh | West Kameng; Changlang |
|  | Assam | Nagaon; Kamrup |
|  | Manipur | Thoubal; Bishnupur |
|  | Meghalaya | Ribhoi; South Garo Hills |
|  | Mizoram | Kolasib; Lunglei |
|  | Nagaland | Dimapur; Peren |
|  | Sikkim | East District; South District |
|  | Tripura | South Tripura; Dhalai |
| **South** | Andhra Pradesh | Chittoor; West Godavari |
|  | Karnataka | Bellary; Mysore |
|  | Kerala | Wayanad; Kollam |
|  | Tamil Nadu | Dindigul; Thiruvallur |
|  | Telangana | Karimnagar; Medak |
| **Central** | Chhattisgarh | Durg; Rajnandgaon |
|  | Madhya Pradesh | Jabalpur; Mandla |
|  | Maharashtra | Ahmednagar; Nandurbar |
| **West** | Gujarat | Anand; Chota Udepur |
|  | Goa | North Goa; South Goa |

**Supplementary Table S2:** ICDS-SNP Nutrient Standards (2012 and 2023) and corresponding Age-Specific ICMR requirements for foods provided to 36-72 month old children

| Nutrient | ICDS-SNP Standard (2012 | *ICMR References used in study (1/3^rd^ EAR)** | ICDS-SNP Standard (2023 Revision) |
| --- | --- | --- | --- |
| Energy (Kcal) | 500 |  | 400 |
| Protein (g) | 12-15 |  | 15-20 |
| Fat (g) |  | 11-14† | 15-20 |
| Calcium (mg) |  | 150 | 300 |
| Iron (mg) |  | 2.6 | 8 |
| Zinc (mg) |  | 1.2 | 3.6 |
| Folate (µg) |  | 37 | 80 |
| Vitamin A (µg RAE) |  | 80 | 100 |
| Vitamin B₆ (mg) |  | 0.3 | 0.3 |
| Vitamin B₁₂ (µg) |  | 0.7 | 0.5 |

*ICMR reference values correspond to one-third of the age-specific Estimated Average Requirements (EAR) and were applied only where programme standards unavailable.

†Fat reference is based on ICMR-recommended fat-energy ratio of 25-35% for children, converted to absolute values for meals providing 400-500 Kcal .

**Supplementary Table S3: Daily nutrient content of foods provided through the ICDS-Supplementary Nutrition Programme to children aged 36-72 months across 27 States and Union Territories in India evaluated against the revised 2023 nutrient standards (Median (IQR))**

| **State** | **Energy^a^ (Kcal)** | **Protein^b^ (g)** | **Fat^c^**  **(g)** | **Calcium^d^ (mg)** | **Zinc^d^**  **(mg)** | **Iron^d^**  **(mg)** | **Folate^d^**  **(µg)** | **Vit A (RAE)^d^**  **(µg)** | **Vit B6^d^**  **(mg)** | **Vit B12^d^**  **(µg)** |
| --- | --- | --- | --- | --- | --- | --- | --- | --- | --- | --- |
| Andhra Pradesh^efg^ | 505.31  (100.04) | 15.27 (1.99) | 13.37 (7.71) | 277.45 (118.42) | 2.3  (0.37) | 5.89 (2.34) | 54.66 (33.43) | 318.11  (263.2) | 0.3  (0.1) | 0.82  (0.14) |
| Arunachal Pradesh^e^ | 482.52  (93.61) | 13.56 (5.21) | 6.98  (5.65) | 37.24  (35.37) | 2.18 (0.84) | 6.48 (1.72) | 34.77 (42.33) | 4.9  (6.28) | 0.19  (0.09) | 0.1  (0.01) |
| Assam^e^ | 676.62  (71.55) | 16.79 (5.18) | 17.48 (3.13) | 171.16 (44.98) | 2.69  (0.8) | 7.15 (2.24) | 52.01 (32.51) | 25.36  (41.97) | 0.22  (0.07) | 0.4  (0.24) |
| Bihar | 519.18 (106.09) | 17.97 (4.84) | 11.97 (6.54) | 101.94 (49.65) | 2.8  (0.72) | 4.86  (1.8) | 124.13 (53.88) | 30.94  (30.13) | 0.34  (0.13) | 0.0  (0.0) |
| Chhattisgarh^eg^ | 416.74 (130.43) | 12.35 (7.73) | 8.11  (9.54) | 75.37  (82.97) | 1.84 (1.14) | 6.26 (4.62) | 70.68 (65.73) | 39.41  (66.77) | 0.17  (0.12) | 0.04  (0.01) |
| Goa | 636.25 (135.32) | 20.59 (5.48) | 33.62 (15.28) | 198.88 (131.71) | 3.41 (1.26) | 7.06 (5.66) | 114.25 (49.81) | 3.12  (2.22) | 0.36  (0.13) | 0.0  (0.0) |
| Gujarat | 498.73 (150.13) | 11.75 (4.41) | 14.31 (7.21) | 52.46  (28.9) | 2.57 (0.92) | 3.91 (1.74) | 43.48 (34.13) | 18.66  (28.71) | 0.32  (0.09) | 0.0  (0.0) |
| Haryana^e^ | 622.87 (209.28) | 15.61 (5.91) | 16.24 (10.15) | 79.91  (45.17) | 3.01 (1.21) | 6.36 (2.25) | 89.09 (58.06) | 35.79  (44.11) | 0.32  (0.13) | 0.02  (0.03) |
| Himachal Pradesh | 469.09 (117.97) | 10.61 (4.12) | 19.11 (10.85) | 84.56  (49.28) | 1.55  (0.6) | 2.74 (1.02) | 57.69 (39.26) | 8.92  (12.82) | 0.17  (0.09) | 0.0  (0.0) |
| Jammu & Kashmir^e^ | 510.76 (100.42) | 10.48  (0.7) | 9.67  (7.61) | 23.86  (13.92) | 1.67 (0.07) | 4.77 (0.77) | 20.78 (17.82) | 1.19  (1.02) | 0.15  (0.03) | 0.07  (0.02) |
| Jharkhand | 500.95 (227.73) | 17.19 (7.15) | 9.8  (4.01) | 55.33  (28.49) | 2.64 (1.13) | 4.46 (1.92) | 56.7 (37.37) | 58.39  (27.57) | 0.29  (0.12) | 0.0  (0.0) |
| Karnataka^ef^ | 425.21  (84.03) | 13.02 (3.13) | 9.94  (5.01) | 90.67  (70.34) | 1.84 (0.45) | 4.56  (1.4) | 38.58 (23.74) | 64.54  (50.31) | 0.25  (0.07) | 0.23  (0.07) |
| Kerala^e^ | 494.61 (136.27) | 14.14 (4.76) | 9.5  (6.49) | 83.81  (50.71) | 2.11 (0.83) | 3.57 (1.43) | 62.25 (24.59) | 81.43  (129.46) | 0.33  (0.13) | 0.02  (0.05) |
| Madhya Pradesh | 347  (62.37) | 10.13 (2.16) | 7.63  (5.06) | 52.76  (53.47) | 1.74 (0.29) | 3.12  (1.1) | 41.23 (26.87) | 10.21  (9.5) | 0.2  (0.09) | 0.03  (0.06) |
| Maharashtra | 399.6  (68.87) | 11.7  (2.14) | 16.39 (4.32) | 61.99  (8.79) | 1.86 (0.24) | 4.24  (0.7) | 80.18 (11.62) | 27.38  (5.51) | 0.24  (0.03) | 0  (0) |
| Manipur | 465.5  (231.31) | 15.44  (4.3) | 11.49 (8.97) | 315.54 (160.71) | 2.00  (0.58) | 4.01  (3.5) | 68.01  (27.1) | 93.78  (113.06) | 0.19  (0.08) | 0.4  (0) |
| Meghalaya^e^ | 598.52 (110.24) | 20.42 (4.66) | 3.95  (1.73) | 281.79 (34.74) | 2.36 (0.88) | 10.75 (3.05) | 74.62 (48.61) | 125.98  (7.61) | 0.21  (0.09) | 0.02  (0.03) |
| Mizoram^efg^ | 524.28 (306.01) | 14.31 (7.71) | 24.09 (15.39) | 179.19 (211.65) | 1.5  (0.66) | 9.56  (7.3) | 52.17 (36.25) | 164.04  (157.01) | 0.14  (0.08) | 0.21  (0.37) |
| NCT of Delhi | 366.45 (202.03) | 12.45 (7.04) | 11.74 (7.63) | 59.56  (44.48) | 2.06  (1.1) | 3.76  (2.2) | 85.29 (75.51) | 28.08  (30.68) | 0.21  (0.13) | 0.0  (0.0) |
| Nagaland | 215.5  (128.24) | 4.91  (5.47) | 3.03  (3.34) | 11.85  (10.54) | 0.81 (0.94) | 1.21  (1.4) | 9.54  (11.43) | 0.64  (1.2) | 0.07  (0.06) | 0.0  (0.0) |
| Odisha^e^ | 522.4  (84.64) | 15.39 (3.64) | 6.04  (4.41) | 58.48  (26.31) | 2.27 (0.47) | 6.08 (0.97) | 56.82 (26.77) | 19.72  (17.08) | 0.35  (0.08) | 0.23  (0.16) |
| Punjab | 458.76 (110.13) | 10.7  (5.0) | 8.12  (4.22) | 158.95 (98.31) | 1.95 (0.59) | 1.73 (1.39) | 13.3  (8.51) | 0.07  (0.08) | 0.21  (0.08) | 0.0  (0.0) |
| Sikkim | 457.33 (153.62) | 14.76 (7.34) | 7.79  (6.34) | 159.66 (171.56) | 2.3  (1.22) | 3.78 (2.47) | 87.71 (45.08) | 13.8  (16.91) | 0.38  (0.32) | 0.38  (0.5) |
| Tamil Nadu^fg^ | 506.92  (51.37) | 13.49 (3.13) | 9.45  (2.1) | 103.64  (28.5) | 2.55 (0.37) | 4.98  (1.00) | 59.6  (20.35) | 78.01  (79.8) | 0.48  (0.11) | 0.04  (0.08) |
| Telangana^fg^ | 374.59 (125.38) | 10.36 (4.02) | 8.13  (5.39) | 27.78  (52.74) | 1.42 (0.78) | 1.63 (1.66) | 40.24 (34.59) | 43.63  (82.57) | 0.16  (0.07) | 0.03  (0.06) |
| Tripura^e^ | 263.93  (74.69) | 9.91  (5.03) | 1.19  (1.37) | 31.76  (30.98) | 1.59 (0.94) | 3.8  (1.99) | 38.44 (48.11) | 1.63  (2.43) | 0.15  (0.09) | 0.06  (0.08) |
| Uttarakhand | 304.46 (191.15) | 13.77 (9.63) | 3.16  (4.87) | 51.03  (43.92) | 2.17 (1.56) | 4.28 (3.14) | 65.34 (54.69) | 8.16  (13.31) | 0.26  (0.21) | 0.0  (0.0) |
| Pooled | 465.3 | 13.6 | 11.2 | 106.9 | 2.12 | 4.85 | 58.95 | 48.37 | 0.25 | 0.11 |
| ICDS (New)* | 400 | 15 - 20 | 15 - 20 | 150 | 1.5 | 3 | 40 | 80 | 0.3 | 0.67 |
| Difference | 65.3 | -1.4 | -3.8 | -43.1 | 0.6 | 1.9 | 18.9 | -31.6 | -0.1 | -0.6 |

The values represent the median (IQR) nutrient provided through SNP meals compared to the 2023 ICDS-SNP guidelines on nutrient standards

Colours indicate the percentage met for each nutrient. Green signifies at least 90% of the requirements are met, yellow suggests that 80-90% requirements are met and red indicates that less than 80% of the requirements are met. In case of energy and fat, red also indicates that the meals provide in excess of 10% of the recommendations.

^a^Energy is considered met if ≥90% of the ICDS standard of 400 Kcal is provided.

^b^Protein is considered met if the minimum ICDS standard of 15g/day is provided.

^c^Fat is considered met if the minimum ICDS standard of 15g/day is provided.

^d^For micronutrients, requirements are considered met if ICDS-SNP 2023 standards are achieved:150 mg calcium, 3 mg iron, 1.5 mg zinc, 40 µg folate, 80 µg vitamin A (RAE), 0.33 mg vitamin B₆ and 0.67 µg vitamin B₁₂.

^e^Anganwadis surveyed in these States used rice fortified with iron (4.25 mg/100g), folic acid (12.5 µg/100g) and vitamin B_12_ (0.125 µg/100g)

^f^Anganwadis surveyed in these States used wheat flour fortified with iron (4.25 mg/100g), folic acid (12.5 µg/100g) and vitamin B_12_ (0.125 µg/100g)

^g^Anganwadis surveyed in these States used Vitamin A-fortified oil (750 µg/litre) in meal preparation

**Supplementary Table S4a: Foods and quantities that reduce nutrient gaps in SNP meals for 36-72 month old children in Karnataka**

| Food Groups | Food items | Quantity (g/ml) | Energy (Kcal) | Protein (g) | Fat (g) | Calcium (mg) | Zinc (mg) | Iron (mg) | Folate (µg) | Vit A (RAE) (µg) | Vit B6 (mg) | Vit B12 (µg) | Price per portion  (Rs) |
| --- | --- | --- | --- | --- | --- | --- | --- | --- | --- | --- | --- | --- | --- |
|  |  |  |  |  |  |  |  |  |  |  |  |  |  |
| Green leafy vegetables | Amaranth leaves | 10 | 3 | 0·3 | 0·1 | 33·0 | 0·1 | 0·5 | 7 | 71·3 | 0 | 0 | 2 |
| Egg | Egg, poultry | 50 | 68 | 6·6 | 4·6 | 24·7 | 0·6 | 0·9 | 24·7 | 0·5 | 0·1 | 0·5 | 6 |
| **Total** |  | 60 | 71 | 7·0 | 4·6 | 57·7 | 0·7 | 1·4 | 31·7 | 71·9 | 0·1 | 0·5 | 8^a^ |
| **Nutrients provided through current SNP^b^** |  |  | 428 | 13·1 | 10·4 | 105 | 1·9 | 4·8 | 44·1 | 76·9 | 0·3 | 0·2 |  |
| **Total nutrients provided (SNP meals+suggested foods)** |  |  | 499 | 20·1 | 15 | 162·7 | 2·6 | 6·1 | 75·7 | 148·7 | 0·4 | 0·7 |  |
| **Recommendation** |  |  | 500 | 12·0 | 13·8 | 150 | 1·2 | 2·6 | 45 | 80 | 0·3 | 0·7 |  |
| **Gap** |  |  | 0 | 0 | 0 | 0 | 0 | 0 | 0 | 0 | 0 | 0 |  |

List and quantities of locally available nutrient-dense foods identified through the optimisation framework for the State of Karnataka, showing their contribution to daily nutrient provision when added to existing SNP meals.

^a^Prices are rounded off to one decimal place.

^b^Nutrients provided through current SNP meals refers to values from Table 1.

**Supplementary Table S4b: Foods and quantities that reduce nutrient gaps in SNP meals for 36-72 month old children in Madhya Pradesh**

| Food Groups | Food items | Quantity (g/ml) | Energy (Kcal) | Protein (g) | Fat (g) | Calcium (mg) | Zinc (mg) | Iron (mg) | Folate (µg) | Vit A (RAE) (µg) | Vit B6 (mg) | Vit B12 (µg) | Price per portion  (Rs) |
| --- | --- | --- | --- | --- | --- | --- | --- | --- | --- | --- | --- | --- | --- |
|  |  |  |  |  |  |  |  |  |  |  |  |  |  |
| Green leafy vegetables | Coriander leaves | 20 | 6 | 0·7 | 0·1 | 29·2 | 0·1 | 1·1 | 10·2 | 63·5 | 0·0 | 0·0 | 0·3 |
| Milk | Cow milk, whole | 200 | 146 | 6·5 | 9·0 | 236·0 | 0·7 | 0·3 | 14·1 | 2·3 | 0·1 | 1·1 | 12 |
| **Total** |  | 220 | 153 | 7·2 | 9·1 | 265·2 | 0·8 | 1·4 | 24·3 | 65·8 | 0·1 | 1·1 | 12^a^ |
| **Nutrients provided through current SNP^b^** |  |  | 349 | 10·2 | 8·1 | 67·5 | 1·7 | 3·3 | 44·3 | 12·4 | 0·2 | 0·1 |  |
| **Total nutrients provided**  **(SNP meals+suggested foods)** |  |  | 502 | 17·4 | 17·2 | 332·7 | 2·5 | 4·6 | 68·6 | 78·1 | 0·3 | 1·2 |  |
| **Recommendation** |  |  | 500 | 12·0 | 13·8 | 150 | 1·2 | 2·6 | 45·0 | 80·0 | 0·3 | 0·7 |  |
| **Gap** |  |  | 0.0 | 0·0 | 0·0 | 0·0 | 0·0 | 0·0 | 0·0 | 1·9 | 0·0 | 0·0 |  |

List and quantities of locally available nutrient-dense foods identified through the optimisation framework for the State of Madhya Pradesh, showing their contribution to daily nutrient provision when added to existing SNP meals.

^a^Prices are rounded off to one decimal place.

^b^Nutrients provided through current SNP meals refers to values from Table 1.

**Supplementary Table S4c: Foods and quantities that reduce nutrient gaps in SNP meals for 36-72 month old children in Maharashtra**

| Food Groups | Food items | Quantity (g) | Energy (Kcal) | Protein (g) | Fat (g) | Calcium (mg) | Zinc (mg) | Iron (mg) | Folate (µg) | Vit A (RAE) (µg) | Vit B6 (mg) | Vit B12 (µg) | Price per portion |
| --- | --- | --- | --- | --- | --- | --- | --- | --- | --- | --- | --- | --- | --- |
|  |  |  |  |  |  |  |  |  |  |  |  |  |  |
| Roots and Tubers | Carrot, orange | 10 | 3 | 0·01 | 0·1 | 3·6 | 0·0 | 0·1 | 2·4 | 56·3 | 0·0 | 0 | 0·2 |
| Milk and Milk Products | Cow milk, whole | 130 | 95 | 4·24 | 5·8 | 153·4 | 0·4 | 0·2 | 9·1 | 1·5 | 0·1 | 0·7 | 7·3 |
| **Total** |  | 140 | 99 | 4·3 | 5·9 | 156·9 | 0·5 | 0·3 | 11·5 | 57·7 | 0·1 | 0·7 | 8^a^ |
| **Nutrients provided through current SNP^b^** |  |  | 402 | 11·7 | 16·7 | 62·5 | 1·9 | 4·3 | 80·8 | 27·8 | 0·2 | 0 |  |
| **Total nutrients provided (SNP meals+suggested foods)** |  |  | 500 | 16·0 | 22·6 | 219·4 | 2·3 | 4·6 | 92·4 | 85·5 | 0·3 | 0·7 |  |
| **Recommendation** |  |  | 500 | 12 | 13·8 | 150 | 1·2 | 2·6 | 45 | 80 | 0·3 | 0·7 |  |
| **Gap** |  |  | 0 | 0·0 | 0·0 | 0·0 | 0.0 | 0.0 | 0.0 | 0.0 | 0·0 | 0·0 |  |

List and quantities of locally available nutrient-dense foods identified through the optimisation framework for the State of Maharashtra, showing their contribution to daily nutrient provision when added to existing SNP meals.

^a^Prices are rounded off to one decimal place.

^b^Nutrients provided through current SNP meals refers to values from Table 1.
